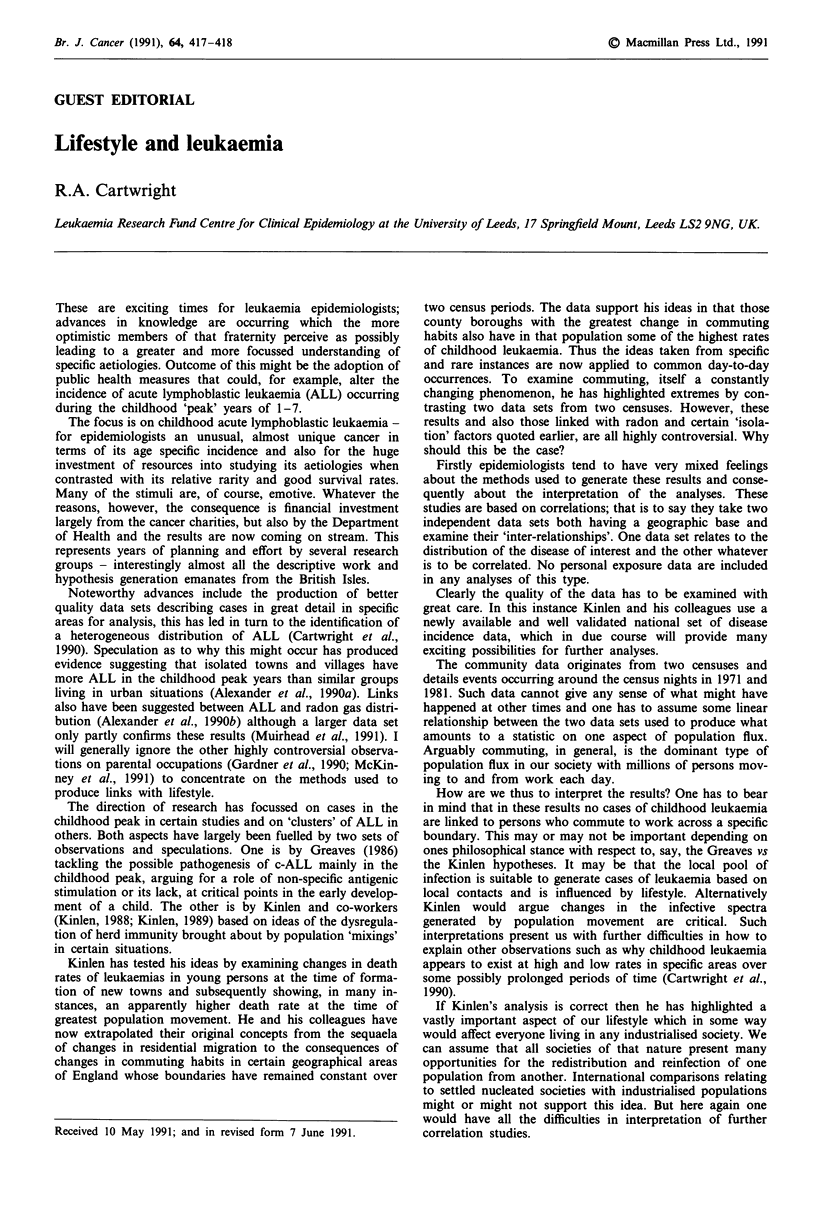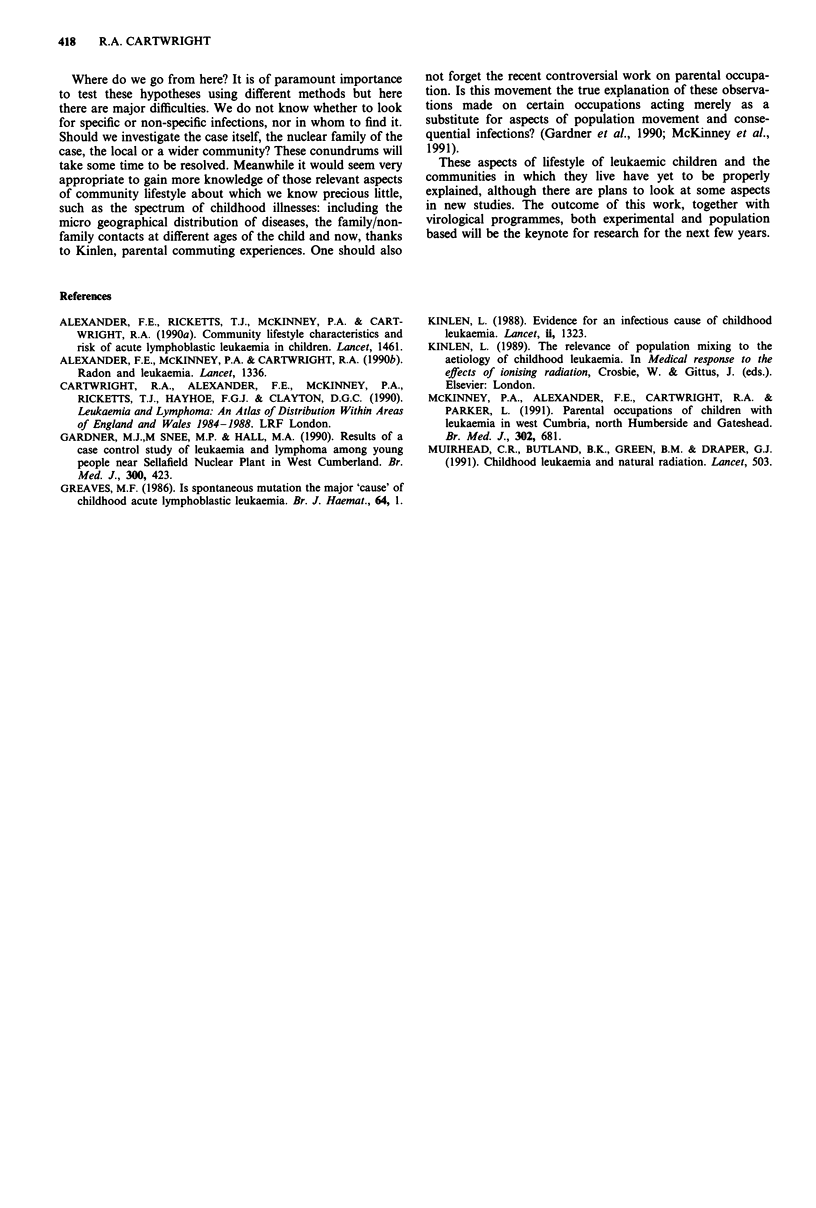# Lifestyle and leukaemia.

**DOI:** 10.1038/bjc.1991.323

**Published:** 1991-09

**Authors:** R. A. Cartwright


 © Macmillan Press Ltd., 1991

GUEST EDITORIAL

### Lifestyle and leukaemia

R.A. Cartwright

*Leukaemia Research Fund Centre for Clinical Epidemiology at the University of Leeds, 17 Springfield Mount, Leeds LS2 9NG, UK.*

These are exciting times for leukaemia epidemiologists; advances in knowledge are occurring which the more optimistic members of that fraternity perceive as possibly leading to a greater and more focussed understanding of specific aetiologies. Outcome of this might be the adoption of public health measures that could, for example, alter the incidence of acute lymphoblastic leukaemia (ALL) occurring during the childhood 'peak' years of 1-7.

The focus is on childhood acute lymphoblastic leukaemia - for epidemiologists an unusual, almost unique cancer in terms of its age specific incidence and also for the huge investment of resources into studying its aetiologies when contrasted with its relative rarity and good survival rates. Many of the stimuli are, of course, emotive. Whatever the reasons, however, the consequence is financial investment largely from the cancer charities, but also by the Department of Health and the results are now coming on stream. This represents years of planning and effort by several research groups - interestingly almost all the descriptive work and hypothesis generation emanates from the British Isles.

Noteworthy advances include the production of better quality data sets describing cases in great detail in specific areas for analysis, this has led in turn to the identification of a heterogeneous distribution of ALL (Cartwright *et al.*, 1990). Speculation as to why this might occur has produced evidence suggesting that isolated towns and villages have more ALL in the childhood peak years than similar groups living in urban situations (Alexander *et al.*, 1990*a*). Links also have been suggested between ALL and radon gas distribution (Alexander *et al.*, 1990*b*) although a larger data set only partly confirms these results (Muirhead *et al.*, 1991). I will generally ignore the other highly controversial observations on parental occupations (Gardner *et al.*, 1990; McKinney *et al.*, 1991) to concentrate on the methods used to produce links with lifestyle.

The direction of research has focussed on cases in the childhood peak in certain studies and on 'clusters' of ALL in others. Both aspects have largely been fuelled by two sets of observations and speculations. One is by Greaves (1986) tackling the possible pathogenesis of c-ALL mainly in the childhood peak, arguing for a role of non-specific antigenic stimulation or its lack, at critical points in the early development of a child. The other is by Kinlen and co-workers (Kinlen, 1988; Kinlen, 1989) based on ideas of the dysregulation of herd immunity brought about by population 'mixings' in certain situations.

Kinlen has tested his ideas by examining changes in death rates of leukaemias in young persons at the time of formation of new towns and subsequently showing, in many instances, an apparently higher death rate at the time of greatest population movement. He and his colleagues have now extrapolated their original concepts from the sequaela of changes in residential migration to the consequences of changes in commuting habits in certain geographical areas of England whose boundaries have remained constant over two census periods. The data support his ideas in that those county boroughs with the greatest change in commuting habits also have in that population some of the highest rates of childhood leukaemia. Thus the ideas taken from specific and rare instances are now applied to common day-to-day occurrences. To examine commuting, itself a constantly changing phenomenon, he has highlighted extremes by contrasting two data sets from two censuses. However, these results and also those linked with radon and certain 'isolation' factors quoted earlier, are all highly controversial. Why should this be the case?

Firstly epidemiologists tend to have very mixed feelings about the methods used to generate these results and consequently about the interpretation of the analyses. These studies are based on correlations; that is to say they take two independent data sets both having a geographic base and examine their 'inter-relationships'. One data set relates to the distribution of the disease of interest and the other whatever is to be correlated. No personal exposure data are included in any analyses of this type.

Clearly the quality of the data has to be examined with great care. In this instance Kinlen and his colleagues use a newly available and well validated national set of disease incidence data, which in due course will provide many exciting possibilities for further analyses.

The community data originates from two censuses and details events occurring around the census nights in 1971 and 1981. Such data cannot give any sense of what might have happened at other times and one has to assume some linear relationship between the two data sets used to produce what amounts to a statistic on one aspect of population flux. Arguably commuting, in general, is the dominant type of population flux in our society with millions of persons moving to and from work each day.

How are we thus to interpret the results? One has to bear in mind that in these results no cases of childhood leukaemia are linked to persons who commute to work across a specific boundary. This may or may not be important depending on ones philosophical stance with respect to, say, the Greaves *vs* the Kinlen hypotheses. It may be that the local pool of infection is suitable to generate cases of leukaemia based on local contacts and is influenced by lifestyle. Alternatively Kinlen would argue changes in the infective spectra generated by population movement are critical. Such interpretations present us with further difficulties in how to explain other observations such as why childhood leukaemia appears to exist at high and low rates in specific areas over some possibly prolonged periods of time (Cartwright *et al.*, 1990).

If Kinlen's analysis is correct then he has highlighted a vastly important aspect of our lifestyle which in some way would affect everyone living in any industrialised society. We can assume that all societies of that nature present many opportunities for the redistribution and reinfection of one population from another. International comparisons relating to settled nucleated societies with industrialised populations might or might not support this idea. But here again one would have all the difficulties in interpretation of further correlation studies.

Received 10 May 1991; and in revised form 7 June 1991.

Where do we go from here? It is of paramount importance to test these hypotheses using different methods but here there are major difficulties. We do not know whether to look for specific or non-specific infections, nor in whom to find it. Should we investigate the case itself, the nuclear family of the case, the local or a wider community? These conundrums will take some time to be resolved. Meanwhile it would seem very appropriate to gain more knowledge of those relevant aspects of community lifestyle about which we know precious little, such as the spectrum of childhood illnesses: including the micro geographical distribution of diseases, the family/non-family contacts at different ages of the child and now, thanks to Kinlen, parental commuting experiences. One should also not forget the recent controversial work on parental occupation. Is this movement the true explanation of these observations made on certain occupations acting merely as a substitute for aspects of population movement and consequential infections? (Gardner *et al.*, 1990; McKinney *et al.*, 1991).

These aspects of lifestyle of leukaemic children and the communities in which they live have yet to be properly explained, although there are plans to look at some aspects in new studies. The outcome of this work, together with virological programmes, both experimental and population based will be the keynote for research for the next few years.

**References**

ALEXANDER, F.E., RICKETTS, T.J., MCKINNEY, P.A. & CARTWRIGHT, R.A. (1990*a*). Community lifestyle characteristics and risk of acute lymphoblastic leukaemia in children. *Lancet*, 1461.

ALEXANDER, F.E., MCKINNEY, P.A. & CARTWRIGHT, R.A. (1990*b*). Radon and leukaemia. *Lancet*, 1336.

CARTWRIGHT, R.A., ALEXANDER, F.E., MCKINNEY, P.A., RICKETTS, T.J., HAYHOE, F.G.J. & CLAYTON, D.G.C. (1990). *Leukaemia and Lymphoma: An Atlas of Distribution Within Areas of England and Wales 1984–1988*. LRF London.

GARDNER, M.J.,M SNEE, M.P. & HALL, M.A. (1990). Results of a case control study of leukaemia and lymphoma among young people near Sellafield Nuclear Plant in West Cumberland. *Br. Med. J.*, **300**, 423.

GREAVES, M.F. (1986). Is spontaneous mutation the major 'cause' of childhood acute lymphoblastic leukaemia. *Br. J. Haemat.*, **64**, 1.

KINLEN, L. (1988). Evidence for an infectious cause of childhood leukaemia. *Lancet*, **ii**, 1323.

KINLEN, L. (1989). The relevance of population mixing to the aetiology of childhood leukaemia. In *Medical response to the effects of ionising radiation*, Crosbie, W. & Gittus, J. (eds.). Elsevier: London.

MCKINNEY, P.A., ALEXANDER, F.E., CARTWRIGHT, R.A. & PARKER, L. (1991). Parental occupations of children with leukaemia in west Cumbria, north Humberside and Gateshead. *Br. Med. J.*, **302**, 681.

MUIRHEAD, C.R., BUTLAND, B.K., GREEN, B.M. & DRAPER, G.J. (1991). Childhood leukaemia and natural radiation. *Lancet*, 503.